# Selective Targeting of Cancer Cells by Copper Ionophores: An Overview

**DOI:** 10.3389/fmolb.2022.841814

**Published:** 2022-03-04

**Authors:** Valentina Oliveri

**Affiliations:** Dipartimento di Scienze Chimiche, Università Degli Studi di Catania, Catania, Italy

**Keywords:** copper ionophores, cuproptosis, cuproplasia, ROS, prodrug, chemotherapeutics, anticancer

## Abstract

Conventional cancer therapies suffer from severe off-target effects because most of them target critical facets of cells that are generally shared by all rapidly proliferating cells. The development of new therapeutic agents should aim to increase selectivity and therefore reduce side effects. In addition, these agents should overcome cancer cell resistance and target cancer stem cells. Some copper ionophores have shown promise in this direction thanks to an intrinsic selectivity in preferentially inducing cuproptosis of cancer cells compared to normal cells. Here, Cu ionophores are discussed with a focus on selectivity towards cancer cells and on the mechanisms responsible for this selectivity. The proposed strategies, to further improve the targeting of cancer cells by copper ionophores, are also reported.

## 1 Introduction

Copper (Cu) is an essential mineral nutrient for all living organisms as it is fundamental in a huge number of biological processes including mitochondrial respiration, iron uptake, antioxidant/detoxification processes (Ruiz et al., 2021). Recently, the role of Cu has also emerged in signaling as a factor that regulates or triggers several biological pathways upon an external stimulus (Li, 2020; Michniewicz et al., 2021). Nevertheless, many connections have been observed between disease statuses and Cu. As for cancer, several studies have reported higher levels of Cu in a variety of malignancies compared to normal tissues. Cu accumulation has been associated with enhanced proliferation and growth, angiogenesis, and metastasis. It is evident that the dyshomeostasis of Cu plays a prominent role in cancer, although researchers debate if it is a cause or a consequence of tumorigenesis. In particular, Cu levels of both serum and tumor tissues have been found significantly altered in patients suffering from different cancers such as breast, thyroid, cervical, ovarian, lung, pancreatic, prostate, gastric, oral, bladder cancers (Basu et al., 2013; Ding et al., 2015; Pavithra et al., 2015; Baltaci et al., 2017; Stepien et al., 2017; Zhang and Yang, 2018; Chen et al., 2019; Aubert et al., 2020; Saleh et al., 2020; Michniewicz et al., 2021). There is some evidence that Cu could have a role in the etiology, severity, and progression of cancer disease (Ishida et al., 2013; Shanbhag et al., 2021). This hypothesis is, for example, supported by the enhanced incidence of hepatocarcinoma in Wilson’s disease patients, the correlation between the stage and Cu levels in colorectal and breast cancer, the link between Cu exposure, pancreatic and prostate cancer (Gupta et al., 1993; Sharma et al., 1994; Ishida et al., 2013; Gunjan et al., 2017; Vella et al., 2017). Moreover, some mechanisms, involved in Cu-dependent growth and progression of tumors, have been recently found and summarized elsewhere (Lelièvre et al., 2020; Ge et al., 2021; Ruiz et al., 2021). Cu is also able to promote angiogenesis that is essential for tumor progression and metastasis. In particular, mounting evidence suggests that Cu can activate many angiogenic factors, such as angiogenin (hAng), vascular endothelial growth factor (VEGF), fibroblast growth factor 1 (FGF1), and interleukin 1 (IL-1), etc. Moreover, Cu can stabilize nuclear hypoxia-inducible factor-1 (HIF-1), therefore further augmenting the expression of the proangiogenic factors (Shao et al., 2019; Lelièvre et al., 2020; Li, 2020). Therefore, Cu alone or bound to ligands promotes angiogenesis in different *in vitro* and animal models whereas Cu chelation suppresses this process.

Since the key role exerted by Cu in the genesis, severity, and progression of cancer, it could be a vulnerable point to target for arresting cancer development (Shanbhag et al., 2021). Conventional cancer therapies suffer from severe off-target effects because most of them target critical facets of cells that are generally shared by all rapidly proliferating cells. Future goals of new therapeutics should aim to increase the selectivity reducing side-effects, overcome cell resistance, and finally, target cancer stem cells. Some metal-binding compounds have shown promise in this direction. These systems are finely summarized in a recent paper (Steinbrueck et al., 2020).

**FIGURE 1:**
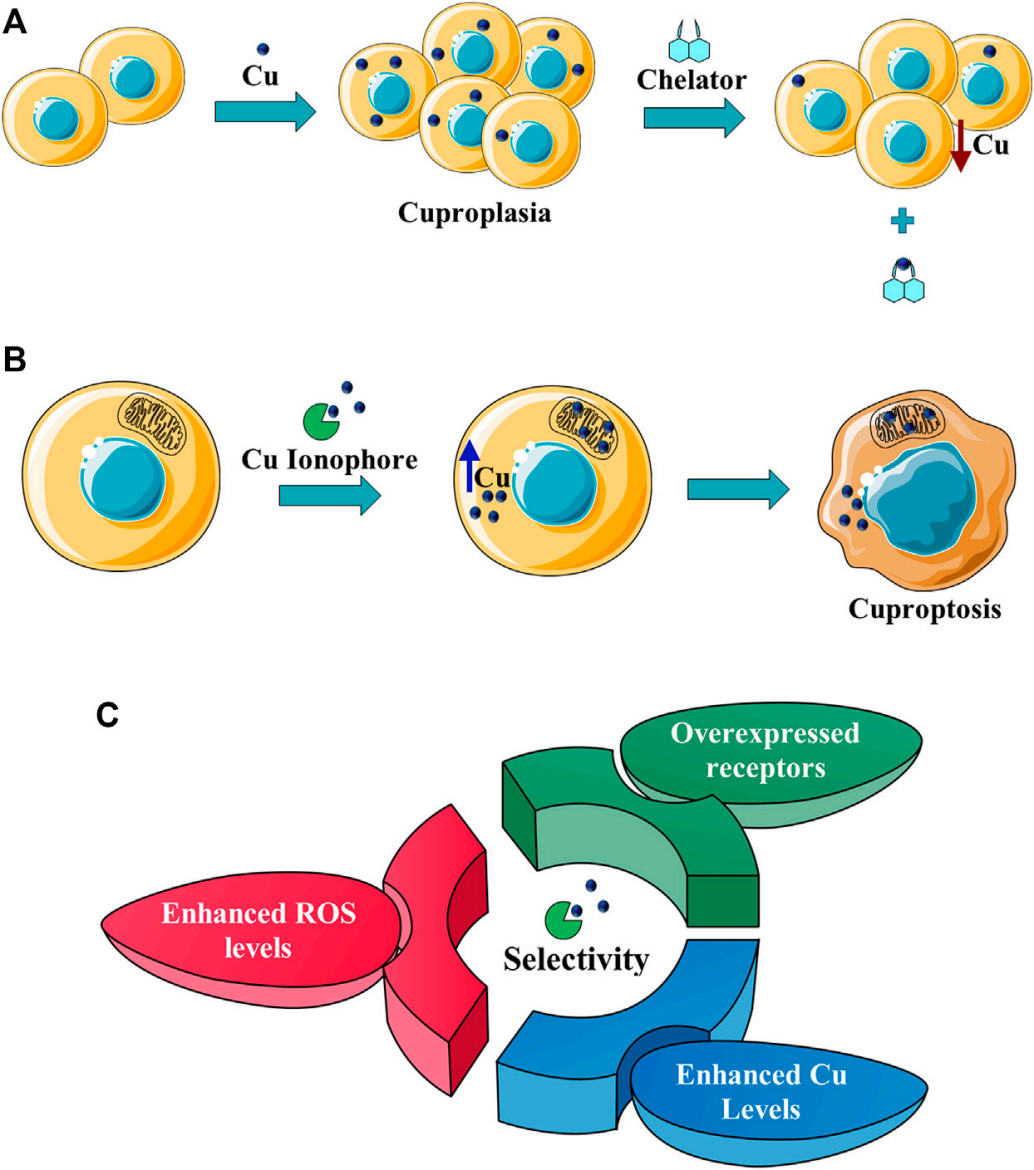
**(A)** Cu chelators inhibit cuproplasia (a Cu-dependent cellular proliferation), decreasing the intracellular Cu concentration; **(B)** Cu ionophores induce cuproptosis, increasing the intracellular (in particular in mitochondria) Cu levels and leading to cell death; **(C)** the intrinsic selectivity of Cu ionophores results from different factors: the increased Cu levels in cancer tissues, the enhanced levels of ROS species that make cancer cells more susceptible to a further increase of oxidative stress, the targeting of receptors overexpressed in cancer cells.

Here, Cu ionophores are discussed with a focus on the selectivity towards cancer cells, the mechanisms responsible for the selectivity (Figure 1), and the strategies proposed to further increase the targeting of cancer cells by copper ionophores.

## 2 Copper Ionophores

Cu metal-binding compounds have a huge potential for the treatment of cancer as well discussed in the review written by Steinbrueck et al. (2020). However, this field is at an early stage of development. In particular, the lack of selectivity is one of the major challenges in this area. Therefore, new Cu-binding compounds able to target selectively cancer cells are highly sought-after. When we refer to Cu-binding compounds, different classes of Cu ligands are included such as Cu chelators and Cu ionophores. Cu ionophores differ from Cu chelators for their mechanism of action as reported elsewhere (Oliveri, 2020; Ge et al., 2021). In particular, Cu chelators inhibit cuproplasia, a Cu-dependent cellular proliferation, whereas Cu ionophores induce cuproptosis (Figure 1). This last term defines Cu-dependent cytotoxicity (with a unique mechanism) leading to cell death (Tsvetkov et al., 2019).

Many different classes of Cu ionophores have been pursued as anticancer agents to promote cuproptosis, including dithiocarbamates, bis(thiosemicarbazone) ligands, 8-hydroxyquinolines (HQs), flavones, etc (Denoyer et al., 2015; Hunsaker and Franz, 2019; Lelièvre et al., 2020; Li, 2020; Ge et al., 2021). The structure of the Cu ionophores mentioned in this paper is reported in Table 1. The most known and studied dithiocarbamates as anticancer ionophores are pyrrolidine dithiocarbamate and diethyldithiocarbamate (DTC), which is the active form of the better-known disulfiram (DSF). DSF is an FDA-approved aldehyde dehydrogenase inhibitor for the treatment of alcoholism, and it has been effectively used for over 60 years. DSF, however, possesses other biological activities and there is been growing attention in repurposing DSF as an anticancer agent (Ekinci et al., 2019; Kannappan et al., 2021). This interest arises from the low cost, high availability, safety profile, and anticancer activity of DSF. In the last years, DSF has demonstrated to act as an anticancer agent against a variety of cancer cell lines (Allensworth et al., 2015; Hassani et al., 2018; Park et al., 2018; Li et al., 2020b). Moreover, numerous studies have shown that the administration of DSF with Cu significantly increases its anticancer action because the active form of DSF is the Cu complex of DTC. The toxicity of DSF seems to be strictly related to the intracellular accumulation of Cu, promoted by DSF (Cen et al., 2004). The mixture Cu-DSF has been tested as an adjuvant in combination with known chemotherapeutic drugs such as cisplatin, temozolomide, gemcitabine, doxorubicin, etc (Li H. et al., 2020). Mounting evidence has demonstrated that Cu-DSF can have several targets including ROS levels, the ubiquitin-proteasome system (UPS), nuclear factor-kappa B (NF-κB), NPL4 (Chen et al., 2006; Skrott et al., 2017; Oliveri, 2020; Kannappan et al., 2021). Despite the excellent results of DSF *in vitro* and *in vivo*, clinical studies of DSF in patients with cancer were not successful. This disappointing outcome could be related to the rapid degradation of DSF and its active metabolite or a different route of administration of DSF and Cu. The chemistry, pharmacokinetics, pharmacodynamics of DSF are comprehensively summarized by Kannappan et al. (2021). So, here, only some aspects related to selectivity, are discussed. Cu-DSF has shown to preferentially target cancer cells compared to normal cells and can also target cancer stem cells (CSC) (Yip et al., 2011; Hothi et al., 2012; Liu et al., 2016; Xu et al., 2017; Sun et al., 2020; Serra Id et al., 2021). Just to give a few examples, Cu-DSF was strongly cytotoxic to leukemia stem-like cells in a dose-dependent manner, while it did not affect normal hematopoietic progenitor cells (Xu et al., 2017). This facet may be correlated to the ability of cancer cells to accumulate Cu but also the oxidative susceptibility of cancer cells. Indeed, the evaluation of ROS levels in leukemia stem-like cells highlighted that the administration of Cu-DSF led to a remarkable intracellular increase of ROS and the triggered apoptosis could be reversed by the antioxidant N-acetyl-cysteine (NAC). The involvement of ROS has also been implicated in the toxic selectivity shown by Cu-DSF versus malignant prostate cancer cells compared to healthy cells (Safi et al., 2014). In this study, a significant increase of intracellular ROS was detected upon the treatment with Cu-DSF and further proof of the ROS-mediated action of Cu-DSF was observed when the administration of free-radical scavengers suppressed the apoptosis induced by Cu-DSF cotreatment (Safi et al., 2014). Elevated ROS levels are a hallmark of human prostate cancer and different studies have demonstrated that oxidative stress plays a role in the progression of prostate cancer (Kumar et al., 2008; Udensi and Tchounwou, 2016). Another interesting study supports the hypothesis that the selectivity shown by DSF arises from differences in the redox biochemistry of Cu in cancer vs. normal cells (Falls-Hubert et al., 2020). Cu-DSF (50–150 nM DSF and 15 μM CuSO_4_) was selectively toxic to H292 NSCLC cells compared to normal human bronchial epithelial cells (HBEC). In this study, Cu uptake was determined to be higher in cancer cells than in normal cells. As a consequence, increased redox reactions of Cu and higher oxidative stress characterized cancer cells compared to healthy cells. Moreover, hypoxia, which is one of the hallmarks of solid tumors, can cause the accumulation of Cu increasing the influx and decreasing the efflux of Cu. In turn, this aspect seems to be associated with the Cu metabolism as the authors observed that the overexpression of ATP7B caused a diminished accumulation of Cu (Falls-Hubert et al., 2020). Targeting chloride channel-3 (ClC-3), a member of the chloride channels superfamily, is another mechanism proposed to explain the selective action of Cu-DSF versus cancer cells (Xu et al., 2019). Cu-DSF can trigger apoptosis through the activation of ClC-3 that is overexpressed in cancer cells compared to normal cells. The overexpression (induced also by the treatment with Cu-DSF) could justify the promotion of cell apoptosis of nasopharyngeal carcinoma CNE-2Z cells (EC_50_ = 0.32 μM) whereas Cu-DSF did not have the same effect in nasopharyngeal epithelial NP69-SV40 T cells (EC_50_ = 1.5 μM) (Xu et al., 2019). Overall, the selectivity, shown by Cu-DSF, may arise due to diverse factors and targets as observed for other Cu ionophores (Figure 1).

**TABLE 1:** The abbreviations and the structures of Cu ionophores mentioned in the paper.

Abbreviation	Structure	Abbreviation	Structure
ATSM	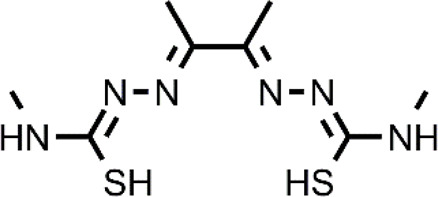	GTSM	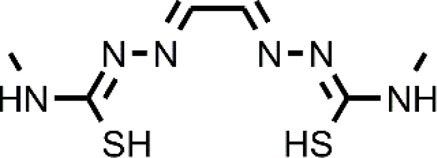
CQ	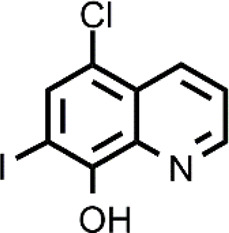	HF	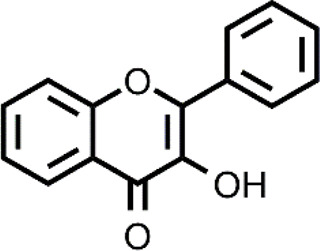
DPy	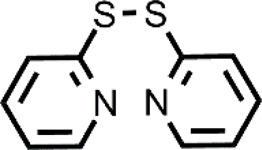	PBT2	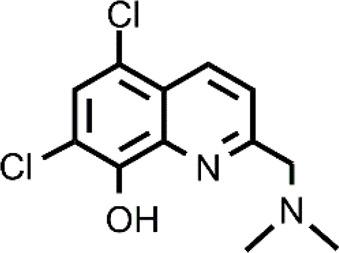
DSF	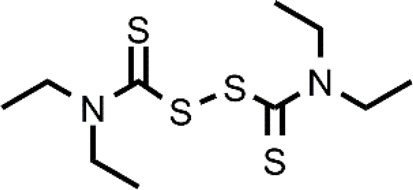	PDTC	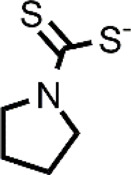
DTC	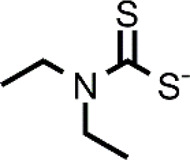	PL-I	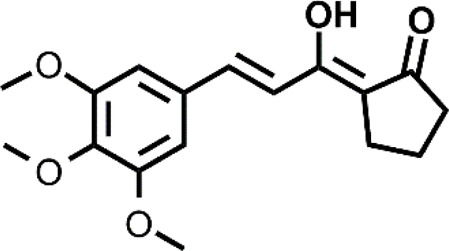
ES	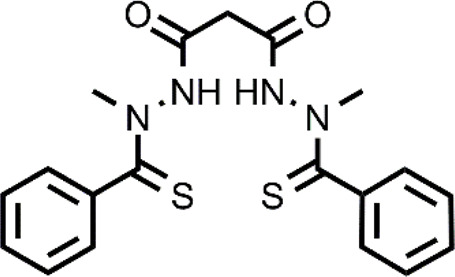	SIH-1	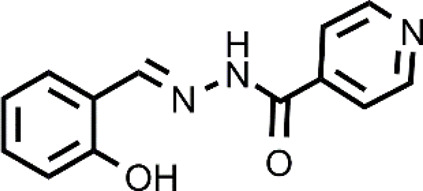

Bis(thiosemicarbazone) analogs are among Cu ionophores that have shown anticancer activity. Diacetyl-bis(N4-methylthiosemicarbazone) (ATSM) and glyoxal-bis(N4-methylthiosemicarbazone) (GTSM) have proven to be effective as anticancer agents. Although these compounds have a similar structure, their mechanism of action is different. SAR studies have indicated that the diimine skeleton of bis(thiosemicarbazone) must not have substituents to exert a high antiproliferative activity. This is the case of GTSM whereas ASTM presents two methyl groups on the diamine backbone that decrease the reduction capacity of Cu-ATSM (Donnelly et al., 2012). Cu-ATSM and Cu-GTSM have been investigated as anticancer agents in prostate cancer cells *in vitro* and *in vivo* (Cater et al., 2013). Cu-GTSM (LD_50_ = 1.5 μM) was more effective than Cu-ATSM (LD_50_ = 7 μM) in killing cancerous prostate PC3 cells *in vitro*. Moreover, Cu-ATSM and Cu-GTSM were also active against human prostate hyperplastic and carcinoma cell lines with different expression patterns whereas they did not affect human primary prostate epithelial cells (PrEC). Finally, the concomitant administration of the physiological concentration of Cu (20 μM) to the culture medium significantly increased the activity of Cu-GTSM (LD_50_ = 150 nM). The authors elegantly demonstrated with a metal responsive element-luciferase reporter that Cu-GTSM can use the extracellular Cu to increase the intracellular Cu level whereas Cu-ATSM cannot (Cater et al., 2013). This different behavior is certainly related to the inability of Cu-ATSM to release Cu in a reducing environment while GTSM can cause the intracellular accumulation of Cu, transporting the ion across the membrane and releasing it. Cu-GTSM was also able to suppress prostate cancer in a transgenic adenocarcinoma mouse prostate (TRAMP) model. It has been hypothesized that the selectivity shown by bis(thiosemicarbazones) seems to be due to the higher content of Cu that is present in cancer tissues compared to healthy tissues. However, a more in-depth study on the targets of Cu-GTSM provides further information and better explains the observed selectivity (Denoyer et al., 2016). The obtained data associate the selectivity shown by Cu-GTSM to the ROS susceptibility of cancer cells as in the case of other Cu ionophores. In particular, TRAMP cells (TRAMP-C1) have high ROS levels together with a decreased glutathione (GSH, reduced form), making them susceptible to the action of Cu ionophores.

Numerous clinical trials have been performed using N^′1^,N^′3^-dimethyl-N^′1^,N^′3^-bis(phenylcarbonothioyl) propanedihydrazide, elesclomol (ES), a well-known Cu ionophore, for the treatment of different types of cancer. Unlike other Cu ionophores, ES has not been repurposed but was discovered upon a high-throughput screening of a compound library and a SAR study against human sarcoma cell lines (Babak and Ahn, 2021). As for the mechanism of action of ES, the induction of oxidative stress that leads to cancer cell apoptosis is the generally accepted mechanism of action. Nevertheless, a series of studies have highlighted the existence of other potential targets (Hasinoff et al., 2014; Hasinoff et al., 2015). Among these, the mitochondrial enzyme ferredoxin 1 (FDX1) has been recently reported (Wang et al., 2020b). The results of clinical trials have not reported serious side effects upon the administration of ES alone or in combination with other chemotherapeutic agents (O’Day et al., 2009; Monk et al., 2018). A feature that bodes well for the further development of this drug as most of the metal-binding compounds presents a series of side effects related to the perturbation of homeostasis of essential metal ions. ES is selective towards cancer cells, but the mechanisms that lead to this selectivity have not been clarified. It might be hypothesized that analogously to DSF or DTC, ES could target cancer cells exploiting the high levels of Cu or ROS. This hypothesis is supported by the data reported by Nagai et al. (Nagai et al., 2012). In brief, ES increased Cu levels and mitochondrial oxidative stress in the HL-60 leukemic cell line whereas it did not influence their levels in peripheral blood mononuclear cells (PBMCs). Another promising feature of ES is its ability to target resistant cancer cells including cisplatin and proteasome inhibitor resistance. The ID_50_ of ES in cisplatin-resistant cells was in the low nanomolar range and the antioxidant NAC was able to inhibit the effects of ES, indicating a ROS mediated mechanism of selectivity (Wangpaichitr et al., 2009).

Cu ionophores include HQs that have a vast variety of biological applications. The most known compound of the HQ class is 7-iodo- 5-chloro-8-hydroxyquinoline (CQ) that was first used as an antibiotic and more recently has been studied for the repurposing in different diseases ranging from neurodegenerative disorders to cancer (Oliveri, 2020). The anticancer action of CQ is augmented by the co-administration with Cu and different targets of CQ have been identified including proteasome (Daniel et al., 2005; Yu et al., 2009; Cater and Haupt, 2011; Cao et al., 2014). Among Cu-CQ targets, it has been identified XIAP, a protein that inhibits caspases, avoiding apoptosis of cancer cells (Cater and Haupt, 2011). Cu-CQ treatment seems to cause XIAP clearance and this effect was observed only in cancer prostate cells and not in normal prostate epithelial cells, indicating a selective behavior of Cu-CQ. Although CQ has shown this slight selectivity versus cancer cells, CQ (unlike ES) presents severe side effects and has also been associated with neuropathy (SMON) (Mao and Schimmer, 2008). For these reasons, CQ must be targeted to cancer cells selectively. In addition to methods for the safe vehiculation of CQ, the researchers have studied other HQ derivatives that could have a greater efficacy as anticancer agents and reduced side effects. Just a few examples include 5,7-dichloro-2-[(dimethylamino)methyl]-8-hydroxyquinoline (PBT2) and nitroxoline that are more active compared to CQ in inhibiting cancer cell proliferation in the presence of Cu (Jiang et al., 2011; Summers et al., 2020). This improved activity could be related to a different mechanism of action or a different cellular Cu distribution (Summers et al., 2020).

It is worth noting that the prolonged use of Cu-binding compounds including Cu ionophores can perturb the homeostasis of essential metals and therefore cause severe side effects in patients receiving the treatment. Although Cu ionophores have shown an intrinsic selectivity versus cancer cells as discussed above, there is a need to expand their therapeutic window for a safer application. So, recently, the research has focused on developing rational strategies and new treatment modalities to increase the targeting of cancer cells.

## 3 Strategies to Increase the Selectivity of Cu Ionophores

**FIGURE 2:**
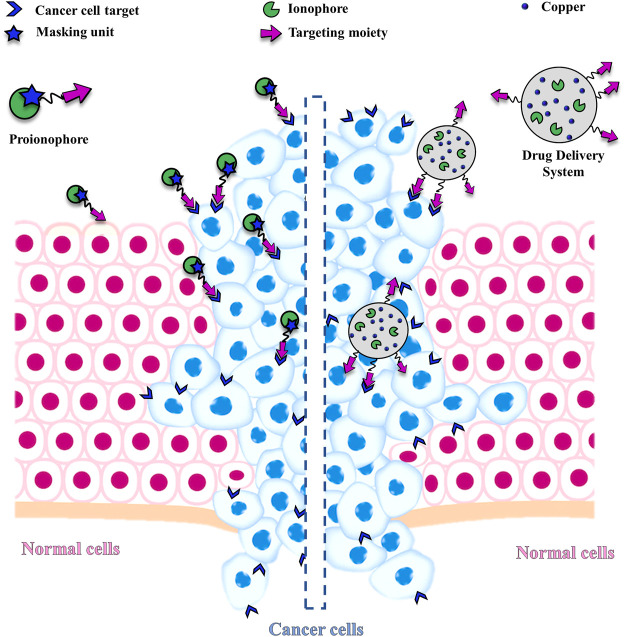
Schematic representation of a generic proionophore and a Nano Drug Delivery System that selectively deliver the ionophore to cancer cells, exploiting a targeting unit.

This challenging goal can be pursued, for example, through the conjugation of targeting units with Cu ionophores (Figure 2). The targeting moieties deliver the ionophore to cancer cells, minimizing side effects. However, the ideal solution would be the concomitant protection of the metal-binding site with the site-specific activation of the metal-ionophore function. In this context, the term proionophore refers to molecules that have to be activated to release the metal ionophore. Another possibility, that has been explored is the application of nano-drug delivery systems that release the ionophore, with or without Cu at the site of action (Figure 2).

### 3.1 Proionophores

What are the features of cancer cells and the strategies that can have been exploited to design Cu proionophores? This paragraph aims to provide the answer to such a question illustrating the advantages and disadvantages of the different methods. Moreover, the papers reported, and some experiments might indicate new avenues for the development of new and effective strategies.

Cancer cells are characterized by an altered ROS production, compared to normal cells (Trachootham et al., 2009). High expression of ROS is caused by the altered metabolism and oncogenic signaling to maintain their malignant phenotype. Therefore, cancer cells are more susceptible to a further increase of ROS above the cellular threshold level (Nogueira and Hay, 2013; Raza et al., 2017). This represents a biochemical feature of cancer cells that can be exploited to develop efficient ROS-mediated anti-cancer strategies such as photodynamic therapy (PDT) and chemodynamic therapy (CDT) (Nogueira and Hay, 2013; Raza et al., 2017). In particular, CDT based on Fenton-like chemistry to convert the less toxic H_2_O_2_ into highly toxic OH has emerged as an effective and innovative anticancer strategy. Nevertheless, the therapeutic efficacy of CDT is limited by the high expression of reducing substances (e.g., GSH) in cancer cells (Wang et al., 2020a). To increase the CDT performance, one strategy has proposed to simultaneously decrease the intracellular levels of GSH. So, the redox dyshomeostasis strategy has been proposed to design new functional molecules and nanomaterials able to disrupt redox homeostasis, augmenting intracellular oxidants and lowering antioxidants (GSH) at the same time (Tang et al., 2019; Li et al., 2020c).

Cu ionophores can also perturb the redox dyshomeostasis, increasing the concentration of the redox-active Cu and exploiting the ROS susceptibility of cancer cells. Some authors have invoked this aspect to explain the selectivity shown by some Cu ionophores, including DSF, CQ, and GTSM, towards cancer cells as discussed above (Safi et al., 2014; Denoyer et al., 2016; Dai et al., 2017; Ji et al., 2018). However, ROS susceptibility can be further exploited through systems activated by GSH or ROS with the concomitant depletion of antioxidants as suggested for CDT (Bao et al., 2018, 2020).

The targeting of cancer cells through oxidative stress-mediating Cu ionophores has also been proposed for other ionophores including 3-hydroxyflavone (3-HF), 2,2′-dithiodipyridine, salicylaldehyde isonicotinoyl hydrazine (Dai et al., 2017; Ji et al., 2018; Zhang et al., 2018). Cu ionophores based on a 3-hydroxy-4-keto group or a keto-enol moiety have shown a GSH-mediated redox mechanism (Dai et al., 2017, 2018). In other words, the release of Cu inside the cells is promoted by the GSH redox action. As a consequence, an intracellular redox imbalance was observed due to the concomitant accumulation of Cu, depletion of GSH, and thereby increase of ROS. In particular, 3-HF, belonging to the family of flavonoids, presented the ability to act as an efficient Cu ionophore and, compared to other similar systems, showed the best cytotoxicity in the presence of Cu against a panel of cancer cells (HepG2, SKOV3, A549) (Dai et al., 2017). Similar results were also obtained for a β-diketo analog of piperlongumine, PL-I, and salicylaldehyde isonicotinoyl hydrazine (SIH-1) (Dai et al., 2018; Ji et al., 2018). The authors managed to demonstrate through a series of elegant experiments that the release of Cu was GSH-dependent for all the last cited ionophores. In brief, the accumulation of Cu caused by the co-treatment Cu-ionophore (PL-I or SIH-1) was detected using Atomic Absorption Spectrometer or ICP-MS, the intracellular dissociation of the Cu complex by GSH was demonstrated by a NIR fluorescent Cu^+^ probe that increased its fluorescence upon the Cu-ionophore treatment revealing the intracellular Cu^+^ accumulation in the cells. Further proof was given by the suppression of increased red fluorescence of the probe when cells were pretreated with L-buthio-nine-sulfoximine, a GSH synthase inhibitor. This finding highlighted the prominent role of GSH in the release of Cu.

A mechanism with depletion of GSH can be also observed in the case of the co-treatment with 2,2′-dithiodipyridine (DPy) and Cu (Zhang et al., 2018). Dpy caused the intracellular accumulation of Cu as determined by inductively coupled plasma atomic emission spectroscopy (ICP-AES). So, the mixture Cu-Dpy was highly cytotoxic against cancer cells, including HEPG2, HeLa, SMMC-7721, and A549 cells. On the contrary, Dpy or Cu alone or the simple diphenyl disulfide did not significantly affect the proliferation of cancer cells. Analogously to the other reported ionophores that exploit the ROS susceptibility of cancer cells, Cu-DPy has shown selectivity against cancerous cells since its antiproliferative effect on healthy cells (L02, BEAS-2B, and HEK 293T) was markedly less. The burst of ROS, caused by the couple Cu-Dpy, was confirmed by using the probe 2′,7′-dichlorofluorescein diacetate DCFH-DA under a fluorescent microscope or determined by a flow cytometer. The authors hypothesized that DPy acts as a Cu ionophore. In brief, DPy forms a 1:1 complex with Cu. The complex passes through the cell membrane and the release of Cu is promoted by the intracellular reductants (i.e., GSH) that cleave the disulfide bridge of DPy. Then, Cu induces ROS formation via redox cycling reactions with thiols or other systems, further affecting the redox-regulating systems (the thioredoxin and GSH systems) and leading to the death of cancer cells mediated by oxidative stress (Zhang et al., 2018).

To further increase the cancer selectivity of Cu ionophores, the Zhou group has proposed the use of proionophores that can be activated exploiting the susceptibility to ROS shown by cancer cells (Bao et al., 2018, 2020). Mounting evidence suggests that proionophores could be more selective, reducing side effects that generally are associated with the administration of metal-binding compounds (Oliveri and Vecchio, 2016). 3-HF has been protected with the 2,4-dinitrobenzenesulfonate group to obtain a proionophore (called PHF), which can be activated by GSH to release 3-HF. PHF was preferentially cytotoxic versus the cancer cells (A549, HepG2, and HeLa) compared to its effect on healthy L02 cells. Mechanistic studies supported the following mechanism: the high levels of GSH in cancer cells activate a huge quantity of PHF. Upon the cleavage, 3-HF promotes the stress-oxidative mediated cancer cell death resulting from the accumulation of Cu, ROS burst, and GSH depletion.

**FIGURE 3:**
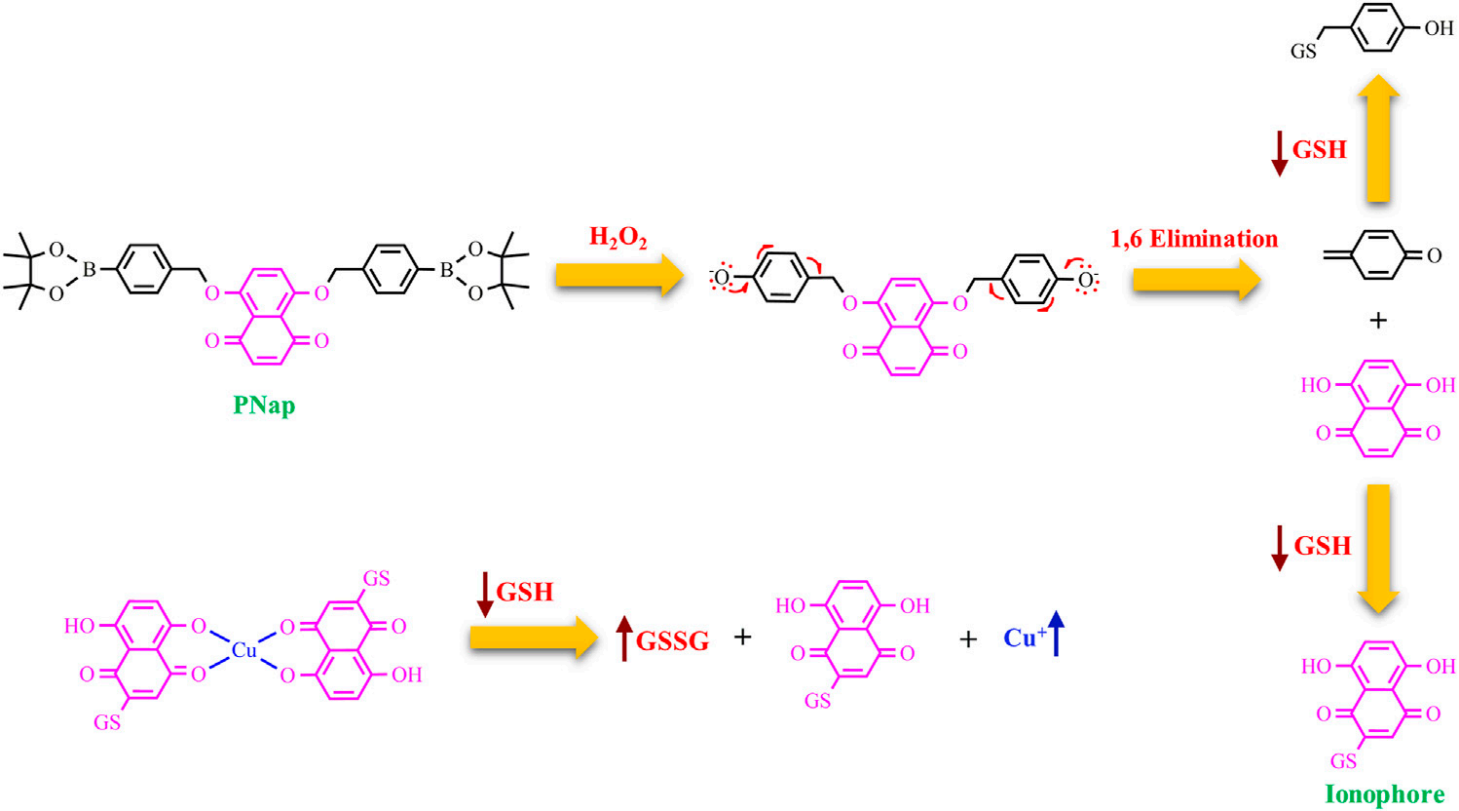
Chemical structure of PNap and the proposed mechanism for the release of Nap upon the action of H_2_O_2_ and a spontaneous 1,6 benzyl elimination. Then, Nap is alkylated by GSH and acts as a Cu ionophore. The arrows highlight the processes, which occur intracellularly (GSH depletion and Cu accumulation).

Zhou group, more recently, has proposed a more sophisticated strategy (Figure 3) with the involvement of H_2_O_2_ in the activation of a proionophore (Bao et al., 2020). This approach is based on the evidence that the levels of H_2_O_2_ are higher in cancer cells than in normal cells, making H_2_O_2_ a good target to develop anticancer proionophores. To validate this approach, the two phenolic hydroxyl groups of naphthazarin, a natural anticancer compound, were protected with the boronate group to obtain PNap (Figure 3). Carbon-boron oxidation is a strategy under strong development to obtain the release of cancer-targeting prodrugs (Maslah et al., 2020). PNap, administrated with Cu, was able to selectively kill human hepatoma HepG2 and cervical carcinoma HeLa cells over human normal liver L02 and umbilical vein endothelial HUVEC cells, respectively. Moreover, the synergistic ability of Cu-PNap in killing HepG2 cells was superior to Cu-PHF. Analogously to PHF, Cu-Nap was less selective in affecting the viability of HepG2 and HeLa cells over L02 and HUVEC cells compared to Cu-PNap (Bao et al., 2020). The mechanism of action proposed is strongly supported by two pieces of evidence: 1) bathocuproine disulfonic acid disodium salt (BCS, a Cu chelator) suppressed the ability of Cu-PNap to kill HepG2 cells; 2) catalase (the enzyme that scavenges H_2_O_2_) also inhibited the effect of Cu-PNap.

H_2_O_2_ activation and the concomitant GSH depletion have also been exploited in the development of a proionophore of DTC (DQ) (Pan et al., 2019). Aryl boronic ester was used as an H_2_O_2_-responsive unit bound to a benzyl moiety that protects the dithiocarbamate group. The authors demonstrated that DQ was activated by H_2_O_2_ to release DTC upon 1,6-benzyl elimination. The results of several experiments indicate that this strategy was successful to increase selectivity. MTT assay showed that DQ (IC_50_>100 μM) was less cytotoxic than DSF against normal NIH 3T3 cells (IC_50_ = 12.5 μM). In the presence of Cu, the IC_50_ value of DQ was 1.4 μM whereas that of DSF was 0.33 μM versus 4T1 cancer cells. Moreover, the addition of exogenous H_2_O_2_ enhanced the IC_50_ of DQ whereas did not affect the cytotoxicity of DSF (IC_50_ = 0.80 μM). The authors correctly highlighted that DSF releases two DTC units and this explains the doubled activity of DSF compared to DQ in the presence of H_2_O_2_. Finally, the value of DSF in the presence of Cu in normal cells was similar to that in cancer cells while the proionophore was selective with an increased IC_50_ of 5 times in normal cells. Furthermore, the measurements of the GSH content revealed a decrease in GSH level upon the treatment with DQ whereas DSF did not influence the GSH/GSSG ratio (Pan et al., 2019). Overall, DQ is able to target selectively cancer cells exploiting the high concentration of H_2_O_2_ and amplifying oxidative stress because of the release of quinone methide, a GSH scavenger.

The site-specific activation of prodrugs can be also obtained by enzyme prodrug therapies (EPT) (Walther et al., 2017). In these approaches, prodrugs are typically designed to achieve a quantitative release of the drug upon the action of a specific enzyme. In particular, one of the EPT strategies consists in exploiting enzymes that: 1) are only found in cancer cells; 2) are overexpressed in cancer cells; 3) change their cellular localization during the cell malignant transformation; 4) change their function in malignant cells (Walther et al., 2017).

**FIGURE 4:**
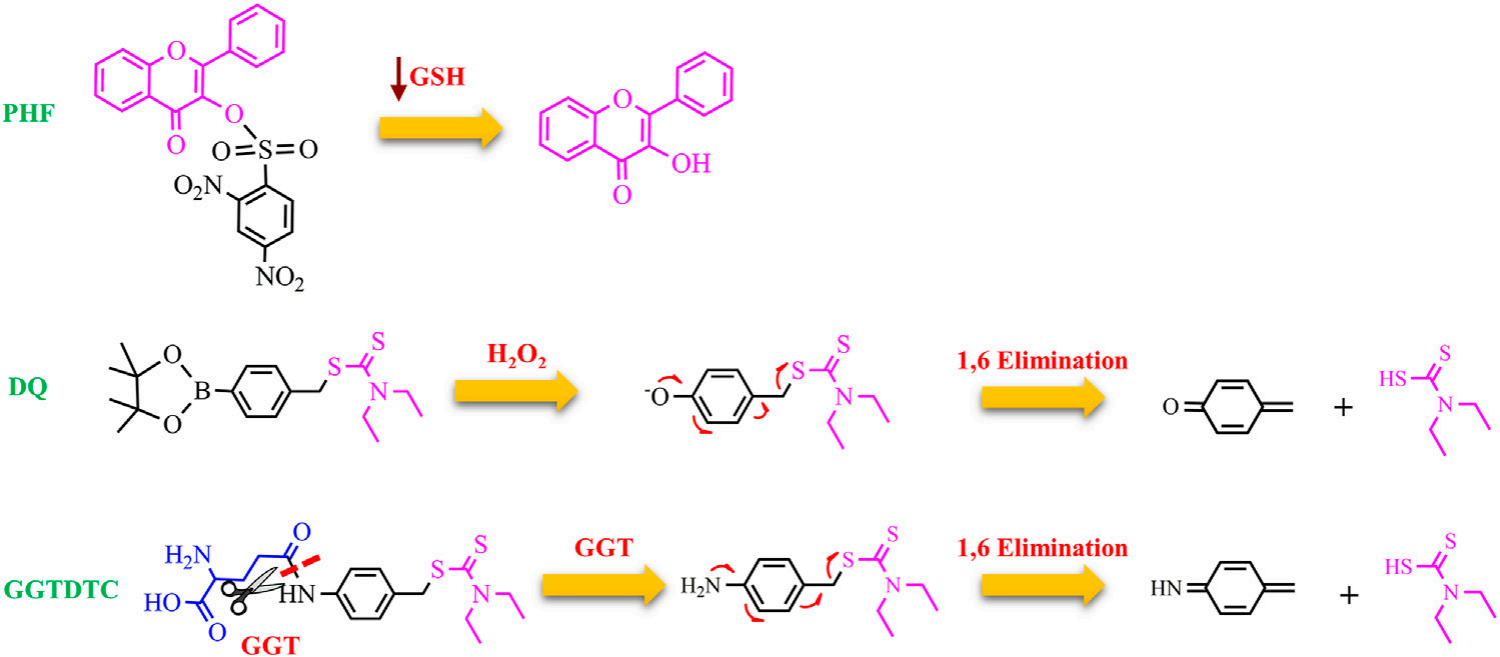
Chemical structure of PHQ, DQ, and GGTDTC and the proposed mechanism for the release of the active ionophores (3-HF and DTC).

Gamma-glutamyl transferase (GGT) is a cell-surface enzyme localized in a wide distribution of tissues including the liver. It is involved in the metabolism of GSH. In healthy cells, GGT only cleaves substrates in ductal fluids whereas GGT, expressed in cancer cells, can hydrolyze GSH and other substrates present the interstitial fluid and blood (Hanigan, 2014). Therefore, GGT plays a key role to provide additional cysteine for the intracellular synthesis of GSH, one of the main components of the cellular redox buffering system. It is therefore not surprising that many cancer cell lines overexpress GGT, and, moreover, high GGT levels seem to correlate with severe outcomes of the disease. In this context, DTC was functionalized with a γ-glutamate residue through a self-immolative p-amino benzyl linker to form GGTDTC (Bakthavatsalam et al., 2018). A series of elegant experiments have shown that: 1) the conjugate was not able to chelate Cu, as demonstrated by calcein competition assays; 2) the conjugate could act as a proionophore because GGT hydrolyzed it, releasing the metal-binding compound; 3) GGTDTC, in the presence of GGT and Cu, formed Cu(DTC)_2_ as demonstrated by mass spectrometry; 4) GGTDTC has a higher affinity for GGT compared to GSH as highlighted by competition experiments in the presence of the colorimetric substrate L-glutamic acid γ-(p-nitroanilide); 5) GGTDTC was significantly cleaved in 22Rv1 cell line, a GGT-positive and aggressive prostate cancer cell line; 6) the GGT inhibitor Acivicin hindered the hydrolysis of GGTDTC; 7) GGTDTC was stable in PWR-1E cells, that do not exhibit GGT activity; 8) the antiproliferative efficacy of GGTDTC correlated with the GGT activity. As a consequence, the IC_50_ of GGTDTC was lower (IC_50_ = 800 nM at 24 h) in cells with high levels of GGT (22Rv1 and LNCaP) and higher in normal prostate PWR-1E cells (IC_50_ = 15 μM at 24 h) (Bakthavatsalam et al., 2018). All these findings support the selective targeting of cancer cells by GGTDTC with the following mechanism of action: GGT cleaves the amide bond, inducing the 1,6 benzyl elimination of the p-amino benzyl linker. The released DTC may chelate Cu in the proximity of GGT-producing cells and lead to the intracellular accumulation of Cu, triggering cancer cell death (Figure 4).

**Figure 5:**
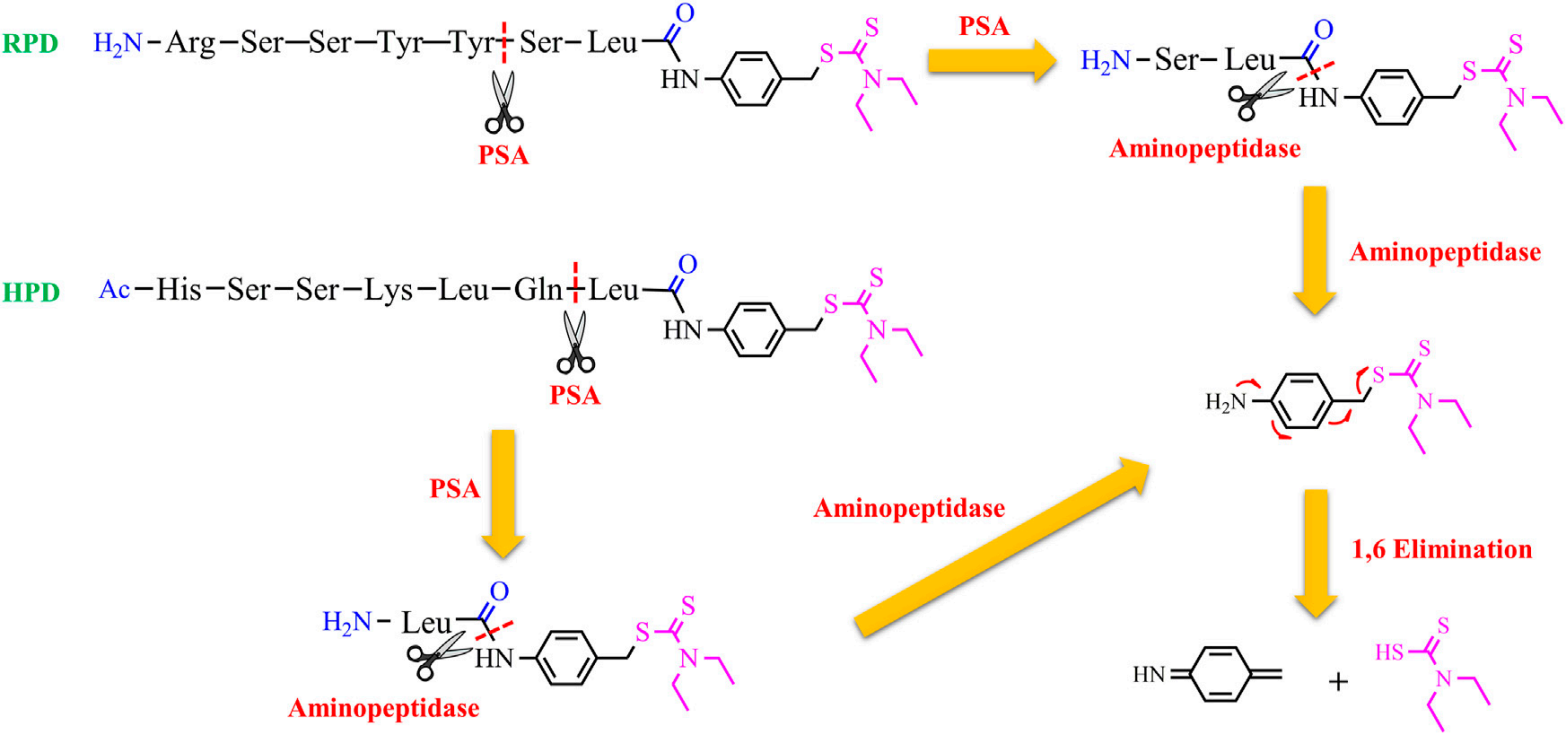
Chemical structures of the proionophores (RPD and HPD) and the proposed mechanism for the release of DTC upon the action of PSA, a generic aminopeptidase, and a spontaneous 1,6 benzyl elimination.

Nevertheless, GGT is not only overexpressed in some aggressive cancer cells but is also upregulated in the liver. So, it is necessary to find more selective targets for cancer cells. Prostate-specific antigen (PSA) could be considered a good choice to target selectively prostate cancer cells. PSA is a 34-kD glycoprotein with a serine protease activity and is overexpressed in prostate cancer cells (Moradi et al., 2019). PSA is active in the prostate, but, it is released as an inactivated form bound to α1-antichymotrypsin in the serum (Hsieh and Cooperman, 2002). This implies that a prodrug, activated by PSA, may release the drug only in the prostate. Taking this into account, two substrates (RSSYYS and HSSKLQ) of PSA were conjugated to DTC through a leucine spacer and a self-immolative p-amino benzyl linker to generate two proionophores RPD and HPD (Bakthavatsalam et al., 2020). The conjugates could have a series of advantages: the thiol group of DTC is masked so 1) it can not bind metal ions, avoiding systemic chelation and the associated side-effects; 2) it does not react with protein and other cellular thiols; 3) the conjugates are more stable than DTC that decomposes in other metabolites with off-targets. It has demonstrated that PSA cleaved HPD between Gln and Leu amino acids whereas RPD was cleaved between Tyr and Ser as confirmed by mass spectrometry studies. Cleavage experiments in lysates indicated that HPD was more selective toward PSA-producing cells and was stable against aspecific proteases. Moreover, the anticancer activity of these proionophores was investigated in androgen-sensitive human prostate adenocarcinoma cells (LNCaP), which express PSA, and androgen-independent PC3 cell lines that do not express PSA (Tai et al., 2011). The cells were treated with HPD or RPD in the presence of Cu. RPD was found to have similar IC_50_ in both cell lines (IC_50_ was 0.77 µM in LNCaP and 1.8 µM in PC3 cells). The D-Ser version of RPD and Cu did not display antiproliferative activity at the same concentrations, confirming that the cleavage is necessary for the activity of RPD. However, the IC_50_ values shown by RPD suggest that the system is non-selectively cleaved by PSA. Indeed, the RSSYYS peptide could be a substrate of other chymotrypsin-like serine proteases. On the other hand, HPD displayed an IC_50_ of 1.4 and 11.2 µM in LNCaP and PC3 cells, respectively. This finding suggests that HPD was more selective than RPD, further confirming the results of the lysate analysis (Bakthavatsalam et al., 2020). Overall, PSA induces the release of DTC, cleaving the peptide. Then, non-specific aminopeptidases remove the final residues to trigger the 1,6-benzyl elimination of the amino benzyl linker leading to the release of DTC (Figure 5). The latter can chelate Cu present in the tumor tissue and induce oxidative stress-mediated cancer cell death. In particular, prostate cancer cells are known to accumulate Cu, a factor that can further improve selectivity.

Glycoconjugation has also been considered an appealing strategy to target cancer cells (Fu et al., 2020). Malignant cells significantly enhance glucose uptake and glycolysis to maintain their high rate of proliferation. This phenomenon, named “the Warburg effect”, is one of the hallmarks of cancer. As a consequence, glucose transporters (i.e., GLUT1) are overexpressed in several human cancers (Szablewski, 2013). Just to give a few examples, anticancer platinum (Pt) systems with appended glucose moieties were active against a panel of cancer cells and accumulated preferentially in tumor cells compared to normal cells (Patra et al., 2016b; Ma et al., 2016). Moreover, an elegant SAR study has demonstrated that the conjugation of the Pt moiety to different positions of D-glucose influences the cellular uptake mediated by GLUT and cytotoxicity of the glycoconjugates (Patra et al., 2016a). In this context, Cu ionophores have been conjugated to glucose and other saccharides to obtain proionophores (Oliveri, 2015). The Cu binding site of these compounds is protected by a glycosidic bond with the sugar unit, so this linkage should be cleaved for the chelation of Cu. The synthesis of different compounds and their evaluation have highlighted the following points: 1) the glucose-HQ compounds were directly hydrolyzed in cancer cells whereas the analogous galactoconjugated systems required the external administration of β-galactosidase; 3) the action of β-glucosidase was fundamental to determine the anticancer effects of the HQ glucoconjugates as demonstrated by the increased IC_50_ in the presence of a β-glucosidase inhibitor; 4) the HQ glucosides released HQs, that induced cuproptosis through a ionophore-mediated mechanism as indicated by the decreased IC_50_ when administrated together with Cu; 5) the presence of different substituents on the HQ moiety influenced the capacity of the glucosides to be cleaved by β-glucosidase, therefore docking studies are required to predict if the enzyme can promote the quantitative release of the HQ derivative from the glycoconjugates. The hypothesized mechanism of action for HQ proionophores lies in the active internalization of these conjugates into cancer cells through specific transporters, the release of the HQ derivative, and the induction of cuproptosis of cancer cells upon the intracellular accumulation of Cu.

Glycoconjugation could be exploited in targeting the asialoglycoprotein receptor (ASGPR) since this strategy has displayed increased tumor accumulation and anticancer activity of the systems conjugated with sugar units recognized by ASGPR (D’Souza and Devarajan, 2015). As for Cu ionophores, this approach has been currently evaluated only for Cu-GTSM for the application in diseases characterized by Cu deficiency (Su et al., 2018). Future development of this strategy could evaluate other applications, for example, in the cancer area.

### 3.2 Nano-Drug Delivery Systems

Another possible approach, which has been explored to target selectively cancer cells with Cu ionophores, involves the use of drug delivery systems (DDSs). Nanoparticle systems preferentially accumulate in cancer tissues rather than healthy ones because of the Enhanced Permeability and Retention (EPR) effect. Moreover, active targeting can be obtained by modifying appropriately the DDS using, for example, cancer-specific targeting ligands or thermosensitive liposomes and mild hyperthermia to reduce undesired off-target effects (Figure 2).

This strategy has been investigated by Gaal et al. that have used a thermosensitive liposomal formulation loaded with Cu and neocuproine (Gaál et al., 2020). The authors also compared this system with analogous thermoresistant DDSs. Even though neocuproine is known as a Cu(I) chelator, the data showed that both DDSs induced intracellular Cu accumulation and *in vitro* and *in vivo* toxicity in BALB/c mice engrafted with C26 cancer cells. Nevertheless, the effect of the thermosensitive formulation was higher compared to the insensitive system. Moreover, mild hyperthermia treatment allowed to reduce the administrated dose when the thermosensitive liposome was used (Gaál et al., 2020).

Moreover, DDSs have been particularly investigated for the selective delivery of DSF to try to overcome the drawbacks associated with its administration (McMahon et al., 2020).

Selective polymeric nanoparticles of DTC (LDNP) were obtained, conjugating lactobionic acid (LBA) and DTC to poly[(2-(pyridin-2-yldisulfanyl) ethyl acrylate)-co-[poly(ethylene glycol)]] (PDA-PEG), endowed of several pyridine-2-thiol groups, via a thiol-disulfide exchange reaction. These polymers forms nanoparticles with a size of 30 nm that are stable in serum, suggesting good stability in blood. Lactobionic acid acted as the targeting moiety because it was recognized by a β-D-galactose lectin receptor overexpressed in some cancer cells, such as liver and ovarian cancers including SKOV-3. The recognition by the receptor remarkably increased the cellular uptake of LDNP compared to analogous nanoparticles without the lactobionic acid as demonstrated by confocal microscopy. This enhanced cellular uptake caused, in turn, higher efficiency in killing cancer cells, in penetrating and dissolving a tumor spheroid model, and, finally, in inhibiting a metastatic ovarian cancer *in vivo*. The selectivity of these systems was demonstrated by low toxicity in normal liver cells and the lack of side effects found in mice treated with LDNP. It has been hypothesized, based on *in vitro* data in presence of GSH, that LDNP releases DTC in the reducing intracellular environment and the active ionophore can bind Cu and lead to cancer cell death. The hypothesis is supported by the evidence that in absence of Cu, all the tested systems significantly failed to kill cancer cells (He et al., 2018). Overall, the data indicated that Cu-LDNP nanoparticles could be a selective and safe tool for the treatment of cancers overexpressing the galactose receptor. The disadvantage of this DDS relies on the different route of Cu administration as Cu are not delivered together with DSF.

Since DSF is rapidly degraded in biological fluids and its activity is increased by Cu, DDSs able to selectively transport DSF at the tumor cells with the concomitant release of Cu could be more advantageous. To validate this hypothesis, different nanoparticle systems have been developed (Chen et al., 2018; Wu et al., 2019; Chang et al., 2020). Li group has proposed metal-organic nanoparticles (MONs) prepared through a method that has been defined as “Stabilized Metal Ion Ligand Nanocomplex (SMILE) technology” (Wehbe et al., 2018). This method provided interesting results, for example, with biomimetic albumin-decorated Cu-DTC MONs (Chang et al., 2020). These particles were prepared by mixing Cu and DTC in the presence of albumin (BSA) using a microfluidic device. The formulation was rather homogeneous with a size inferior to 100 nm. MONs prepared with BSA were stable in serum and could target selectively cancer cells exploiting the EPR effect but also the SPARC receptor, which binds BSA and is overexpressed in cancer cells. As a consequence of the accumulation in the tumor site, the BSA Cu-DTC MONs showed the highest anticancer activity and a good tolerance compared to the other tested controls (Chang et al., 2020). Recent advancement of this DDS uses the proionophore DQ in building the nano delivery system and strengthening the toxicity of DTC with a combination of therapies (Kang et al., 2021). In this system, the activation of DQ is increased by the ROS formed by NIR laser action, Cu is also delivered *in situ* together with DSF. The toxicity of Cu-DSF is exacerbated by the tumor ROS amplification in response to NIR light treatment and, the induction of immunogenic cell death with concomitant GSH depletion (Kang et al., 2021).

Pegylated mesoporous silica nanoparticles doped with Cu and DSF were also tested *in vitro* and *in vivo* with good results. The acidic condition of the tumor environment induced the degradation of the nanoparticles with the concomitant release of DSF and Cu, whereas the nanosystem was stable at physiological pH. Upon the release, DSF reacted with Cu and the triggered Fenton-like chemistry also led to the formation of radical ROS species. This amplified the cytotoxic effects of Cu-DTC as confirmed by the experiments in 4T1 tumor-bearing female BALB/C nude mice. Indeed, the efficacy of the drug delivery system was indicated by the high inhibition rate of 71.4%, compared to DSF alone that did not show chemotherapeutic efficacy (Wu et al., 2019).

Finally, DDSs have also been proposed for CQ or other HQ derivatives but we are far from achieving analogous results to those obtained for DSF (Wang et al., 2013; Weerasuriya et al., 2017; Wehbe et al., 2018; Campos et al., 2020). Nevertheless, the development of a selective DDS for CQ could be a turning point to solve the toxicity problems that led to the CQ withdrawal from the market.

## 4 Conclusion

The discovery of numerous additional pathways that involve Cu and Cu-dependent proteins is proof of the extraordinary progress in the field. In this respect, it suffices to note that two new terms “cuproplasia” and “cuproptosis” have been recently used to indicate unique biological processes triggered by Cu action. In this context, Cu ionophores that could selectively induce cuproptosis may well succeed in overcoming the limits of traditional anticancer drugs. Despite a great number of studies, there is a poor understanding of how Cu ionophores act as selective anticancer agents. Generally, the most accepted theories suggest that Cu ionophores exploit the massive presence of Cu in tumor tissue, alternatively, they take advantage of cancer cell susceptibility due to oxidative stress. Nevertheless, many questions remain regarding these mechanisms and there is the need to address these questions to better exploit their action in arresting cancer progression. However, their intrinsic selectivity can be expanded to obtain a larger therapeutic window. In this context, the results obtained suggested that the most efficient systems are the proionophores that are selectively activated in tumor cells. Most of the studied systems are preferentially accumulated or activated in cancer cells, exploiting a metabolic feature of cancer cells (i.e. ROS susceptibility, glucose avidity, etc). The choice of the targeting unit of the proionophores or nano-drug delivery systems is crucial in designing these systems to deliver the cancer drug exclusively to the cancer tissue. In other words, the next generation of selective Cu ionophores should exploit targeting units that can be recognized only by specific receptors that are exclusively found in a particular type of cancer cells (i.e. prostate or breast cancer cells). Cu proionophores and nano-drug delivery systems are at the early stage of development, but the obtained results show promise for developing innovative therapies that can expand the existing landscape of cancer chemotherapeutics.
